# Indigenous peoples as trustees of forests: a bio-socio-cultural approach to international law

**DOI:** 10.1007/s10784-024-09654-w

**Published:** 2024-12-20

**Authors:** Liliana Lizarazo-Rodriguez, Alice Lopes Fabris, Doreen Montag

**Affiliations:** 1 Brussels School of Governance, Vrije Universiteit Brussel, Brussels, Belgium; 2 Wolfson Institute of Population Health, Queen Mary University of London, London, UK

**Keywords:** Indigenous peoples, Forests, Mother earth, Bio-socio-cultural, Multidisciplinary approaches to international law

## Abstract

Forests are an essential part of Mother Earth within the Earth system. Deforestation is a widespread practice due to systematic land-use change. The international community is concerned, but no instrument explicitly protects forests, which are essential to avoid overshooting planetary boundaries and protect planetary health. Indigenous peoples (IP) and forests have a long-standing relationship, and both are affected by deforestation and biodiversity loss. The intrinsic and existential relation of some IP with forests needs recognition and the establishment of mechanisms to protect their rights, society, and culture, to address the disappearance of these ecosystems. The role of IP in forest governance has been mainly assessed outside international law, from multidisciplinary or interdisciplinary fields. The multidimensional nature of the interaction between IP and forests could explain this phenomenon. This article uses a bio-socio-cultural approach to assess whether protecting the bio-socio-cultural rights of IP, which are intertwined with their ecosystems and territories, is a way to protect forests, and whether this view is consistent with international law. The article presents a multidisciplinary narrative literature review and identifies the main gaps in international law and policy on the protection of forests and IP. It makes three contributions. First, it shows the convergence of scientific evidence that IP are undoubtedly essential actors in the conservation of the ecosystems in which they live. Second, it discusses how this empirical evidence on the pluralist view of forests implies a transnational approach to involving peoples in the governance of their natural resources. This is, IP views on forests should be considered when addressing governance gaps of the Earth system. Third, it assesses how states need to recognise the plurality of their peoples and the need to prioritise the protection of key ecosystems and IP. In the same week that the Conference of the Parties to the Convention of Biological Diversity (CBD-COP16) acknowledged the multi-ethnic nature of the world's populations and recognised people of African descent and IP as key stewards in conservation efforts, this article was accepted for publication. Without this recognition of plural visions, Mother Earth will collapse.

## Introduction

Studies emerging in the XXI Century show that the global impact of economic activities is threatening Mother Earth. The Planetary Boundaries (PB) framework (Rockström et al., 2009) visualises the global environmental limits within which humans can safely operate (Steffen et al., 2015) and how, if humans continue exploiting ecosystems in the same way, the Planet may collapse. Forests are home to the greatest planetary biodiversity, and its degradation affects various PB (Winkler et al., 2021). In 2023, the update of the PB framework alerted that although the boundary on land system change has moderately been crossed, the ones on novel entities, biosphere integrity and climate change have been extensively crossed. They present high risks of triggering dangerous tipping points in the Earth system (Richardson et al., 2023). Solid scientific evidence has shown that vulnerable populations are more affected by the destabilisation of the Earth system. However, the international community has not adopted a global response. Solid scientific evidence also demonstrates that populations intimately linked to highly biodiverse ecosystems, such as Indigenous Peoples (IP)^1^ can play a crucial role alongside transdisciplinary scientific contributions in addressing these global challenges. They are also important actors to determine together with the international community how to integrate a Mother Earth care approach into the Earth system governance.^2^

Although forest preservation and its sustainable use have been included in the United Nations (UN) agenda, no binding treaties have been adopted to protect forests, and to combat deforestation. Furthermore, IP, who are often forest inhabitants, usually do not reach international fora. Frequently, state and non-state actors’ interests have clashed with IP rights and territories, threatening IP’ wellbeing and survival. The interaction between IP and forests has been assessed within diverse fields, mainly from multidisciplinary perspectives that support the implementation of participatory and integrative mechanisms of forest governance, in harmony with the IP cosmovision. From a legal perspective, this is a new field. It is gaining relevance in laws and jurisprudence that recognise rights of Mother Earth. However, this analysis goes beyond seeking the adoption of rules that grant rights: it adds new elements to the ongoing discussion about the need to re-think how plural visions can be heard within the making of international law (IL).^3^

If this interaction has not been a matter of protection in IL, other sciences reveal that it is essential for the viability of both forests and IP that inhabit them. IP interaction with forests is sociocultural, as it relates to social structure, such as gender, age, socioeconomic status, and cultural aspects, such as cosmology. The term ‘bio-socio-cultural approach’ (BSCA) has been used in medical anthropology to understand the relationship between health and disease, social structure and cultural practices, biological processes, addressing and including IP knowledge, perceptions, experiences and practices (Pedersen & Baruffati, 1985; Singer & Erickson, 2013). With increasing understandings on planetary health, One Health and the interrelationship between environment, animals and human health, which are tied to forest and biodiversity protection, it is also used in the context of pandemic research (Radhakrishna, 2023). Environmental conservation and socioecological systems, where knowledge, values and practices of IP are integrated, align with the socio-cultural and bio-cultural dimensions, but not as an integrated framework for analysis (Berkes, 2021; Brondízio et al., 2021). Nirmal et al., (1999, p. 336) use the term “bio-socio-cultural factors” to refer to the interrelationship between culture, biology (environment) and society in relation to Khatan and Waghai Forests, in India. Ethnoecology also employs BSCA, based on the interrelationship between ‘nature’, ‘culture’ and ‘society’ and the need to include local knowledge in sustainable development (Beltrán & Castro, 2014). BSCA’s core idea is that the relationship of IP with forests also has a social aspect (Beltrán & Castro, 2014, p. 131), which goes beyond the bio-cultural rights approach. The bio-cultural rights recognise the environment-culture nexus, but the BSCA recognises the importance of nature for IP and integrates how IP conceptualises ‘nature’ rights within its social organisation. We use and propose a BSCA to the analysis of IP as trustees of forests.

IP cannot be generalised. IP live in diverse places across the world, in urban, semi-urban and rural areas, some in voluntary isolation, some engaging in pastoralist activities, in fishing, hunting etc. The UN Human Rights Council (UNHRC) refers to IP living in forests, isolation, and pastoral areas.^4^ This article refers to IP living in forests, but even among those, distinct groups may have multiple perceptions about forests, practices of engagement and ontological conceptions of Mother Earth. We do not generalise this plurivision but highlight that it is this plurivision that needs to be actively integrated in IL when adopting any measure establishing duties of states on forest protection.

For this review, mainly social science studies have been researched from a conceptual and empirical perspective to encompass the themes conceptualised as BSCA. Their conclusions go beyond the protection of IP as vulnerable groups. They address an integration and empowerment of IP, who can contribute to the protection of forests in a scenario where states seem unable/willing to adopt treaties to control land-use change and prevent deforestation. Furthermore, there is a need for mechanisms to protect IP’ rights and IP’ role as trustees of forests. Some studies have referred to the need of combining justice considerations to the Earth System governance (Kashwan et al., 2020; Rockström et al., 2023). While the term Earth system governance represents ways to manage the planet, the concept of Mother Earth enables an analysis on forest protection as part of a caretaking process aligned with IP’ cosmovision.

Whether states need to adopt a forest convention is a debate that has been addressed by legal scholars. However, the current planetary crises require humanity to go beyond the law in books and acknowledge the scientific evidence showing that integrating IP living in forests in any regulatory or management measure aimed at forest conservation is essential, given the evidence that forests, where IP live, are well conserved. This article proposes to integrate a BCSA to IL on forest protection because scientific evidence shows that IP’ cosmovision and care of forests is a powerful management system that has shown better results than exclusive state-based management of the natural resources. Unveiling the socio-cultural dimension of the interaction between IP society and forests is an important contribution from other social sciences that can guide how IL needs to go beyond the label of IP’ cultural rights and recognise the social dimension of their societies that includes the ecosystems and territories where they live,^5^ which has proven to be very efficient for forest management.

The article has the following sections: first it presents the methodology, followed by a literature review. The article also assesses the gaps in IL regarding forest protection and explains how, IL protecting IP’ rights and their lands and territories,^6^ can enhance forests protection where they inhabit. This is followed by a discussion of the main findings and conclusions.

## Methodology

This article seeks to connect theoretical insights and empirical evidence brought by multiple research fields and see how IL instruments could establish state duties and inform policy choices to integrate IP interaction with forests by understanding and integrating BSCA. First, we conduct a literature review, that we do not claim to be systematic or comprehensive, as it covers multiple fields that have been working on related topics. We mainly focus on peer-reviewed articles and books from the fields of IL in general, and environmental, sustainable development and transnational law in particular, and law and ecology, IP studies, law and criminology, etc. However, from our search, it appears that more than in legal areas, other fields such as environmental justice (EJ),^7^ development studies, ecological economics, philosophy of economics, sociology, anthropology, ethno-biology/ecology, environmental sciences, indigenous studies, Earth system governance and planetary health have robustly contributed from an empirical and conceptual perspective to the understanding of the research problem. The result of the search^8^ unveils that this interaction has mainly been explored from inter or multidisciplinary fields, which often involved empirical studies. Remarkably, legal sources rarely include empirical studies, which limits conclusions about the effectiveness of legal systems. However, not all the publications explain the methodology used. Most of them bring new knowledge by using legal and conceptual analysis, position papers, ethnographies, qualitative case studies, historical and policy analysis, literature review, and quantitative empirical methods. The geographical span is mostly global, and case studies focus mainly on Latin America, Asia, and Oceania. Several databases, namely JSTOR, Google Scholar, HeinOnline, were reviewed to find sources on the interaction between IP and forests. This review reinforces the validity of our contribution that recommends IL, first, to open spaces for the integration of scientific contribution from multidisciplinary approaches when regulating issues that fundamentally require empirical evidence, and second, to integrate IP interests when adopting regulatory measures for forest protection and management that cannot neglect plural views.

Second, the legal analysis was oriented by a gap finder objective, which sought to explore the binding and non-binding norms on forest protection, IP' rights directly related to the subject of this article, i.e. collective land tenure and self-determination, and the bio-socio-cultural interactions between forests and IP. We also mapped relevant policies adopted by international organisations,^9^ and succinctly presented the international jurisprudence on the subject, notably from the UNHRC, the ILO mechanisms^10^ and the Inter-American Court of Human Rights (IACtHR). In the discussion we integrate this law and policy gap analysis and connect to the scientific evidence on BSCA to forests, seeking to propose avenues for integrating a BSCA to IL.

Regarding the limitations, first, the issue of IP and forests encompasses many concepts, national laws and case law. However, the scope of the article is to develop how scientific evidence could support a pluralist vision of forest management and protection within IL by recognising the significant role of IP interaction with nature. Second, ILO Observations are more than 600, so we only consulted those published since 2018 for feasibility reasons. Similarly, UN Permanent Forum on Indigenous Issues (UNPFII) recommendations database contains over 1700 entries. We filtered them by using the keyword "member states" and selected those related to environment and development.

## Literature review

### Global economic challenges and non-conciliatory cosmovisions

Current policies on forest management, forestry and conservation exhibit a dual conception of forest and society. Forests have been exploited as commodities and ecological services (Eikermann, 2015, p. 12–13; Kennedy et al., 2023). Overexploitation has led to systematic land-use change and deforestation that generate multiple environmental harms such as biodiversity loss, water stress, global warming, and pollution. Mainstream sustainable development, environmental economics and international environmental law (IEL) have focused on preventing the exploitation of natural resources from undermining future growth and generations, but overlooked vulnerable communities affected by land-use change (Kronenberg, 2010; Lizarazo-Rodríguez, 2021; Naveed et al., 2022; Stern, 2004). IEL and environmental economics have sought to prevent or manage the cost of environmental degradation, so that sustainable economic growth, or green growth with limited adverse impacts, can keep globalised societies functioning. The approach is criticised from areas such as ecological economics (Ayres, 2008; Muniz & Cruz, 2015). The emergence of climate law aligns with the mainstream concept of sustainable development as it aims at limiting greenhouse gases (GHGs) and not primarily at preventing environmental degradation (Zahar, 2021).

Empirical work has demonstrated that extractives have historically been mainly responsible for the Earth system degradation (De Sa, 2019; Petavratzi et al., 2022). Forests have also been affected by large-scale agrifood activities that also contribute to the release of GHGs, biodiversity loss, water scarcity and deforestation (Lenzen et al., 2012; Springmann et al., 2018). These sectors have also released significant amounts of hazardous materials and waste, and although IL has somehow regulated their use and disposal, this has not prevented mankind from overstepping this boundary (novel entities) (Fernández‐Llamazares et al., 2020; Kennedy et al., 2023; Richardson et al., 2023). However, these economic sectors are not the only concern. Multiple complaints of deforestation and biodiversity loss have been documented against clean energy projects promoted to decarbonise the global economy. Some studies have shown serious impacts they generate in IP’ lands and forests (Avila, 2018; Murgas et al., 2021; Ramirez, 2021; Zárate-Toledo et al., 2019).

Literature as varied as sociology, anthropology, and EJ has documented both empirically and theoretically conflicts that emerge when the value attributed to ecosystems is not unanimously shared (Bauer, 2016; Figuereido & McDonald, 2019).^11^ Some states have deprived IP from their right to manage forests within their territory, seeking to implement projects to engage with value chain activities (Dowie, 2011; Kröger & Lalander, 2016; Neves & Igoe, 2012; Peters, 2018; Zaremberg & Wong, 2018). Likewise, when conservation policies collide with IP rights, literature mainly in areas of sociology and EJ, qualify these conflicts as a reproduction of coloniality, termed ‘continued colonisation’ (Quijano, 2000), ‘green colonisation’ (Domínguez & Luoma, 2020; Kumar, 2010; Paliewicz, 2022; Ybarra, 2018), or ‘green grabbing’ (Fairhead et al., 2012; Franco & Borras, 2019). These practices could be well-intentioned but may also reproduce unequal power relations and geopolitics surrounding access to ecosystem services that have led to endangering forests.

### A plural vision of forests

Multiple studies point to the need for sustainable development policies to prioritise the protection of the biological equilibrium (Raworth, 2017; Robinson, 1993). The PB Framework (Rockström et al., 2009) and Planetary Health (Whitmee et al., 2015) also alert that the disappearance of forests may trigger various tipping points in the Earth system. However, states face the dilemma of preserving forests and ensuring sustainable use of their natural resources. Differences in perspectives regarding forests or the value attributed to forests – forests as an ecosystem, as part of a society or as a commodity – have generated multiple conflicts worldwide.

Ecological economics has looked at the value attached to ecosystems and how valuation can be applied to their management (Taye et al., 2021). However, the economic value of the natural resources is not the only possible value. People also attach intrinsic value to nature, and even keeping well-preserved ecosystems is becoming an emerging economic value (Davidson, 2013, p. 173; Krutilla, 1967). An intermediate approach attributes an ‘existence’ (Davidson, 2013) or socio-cultural value to ecosystems to highlight how some people value ‘nature’ beyond its economic or intrinsic value. A systematic review of the literature shows that these multiple views have evolved, with different conceptualisations, but with a growing consensus on the need for this plural vision to reach policy-making spaces (Pascual et al., 2023). In addition to the economic and intrinsic value, the intermediate option (existence value or BSCA) is increasingly recognised as essential to protect the planet (Gupta et al., 2024; Pascual et al., 2023; Zurba et al., 2024).

The relevance of this interconnection among IP territories, ecosystems, and particularly forests, has been shown by anthropological studies that have shed light, based on empirical evidence, on the long-standing connection of IP with their surroundings, the forests they live in, with whom they interact as much as with other species, and on conceptions of nature and society across the Amazonia (Kohn, 2013; Rival, 2021; Viveiros de Castro, 2014). Ethnographic work on Huaorani people’s perception of forests, builds on earlier literature on symbolism and social relations between humans and their environment, including trees (Rival, 1993). She explains how Huaorani people understand growth and aging in relationship to specific tree growth and aging as part of a life cycle beyond individuals, at the level of the whole society. Rival’s book (2021) the ‘Social Life of Trees’ presented theoretical, ethnographic, and political ecology cases, exemplifying how social relations with the biosphere become cultural. While these works look more at the symbolic side of trees, Kohn’s (2013) ethnography argues for anthropological research to endeavour outside the human to the study of ecological selves, encompassing non-human entities. Based on ethnographic research among Runa people in Ecuador, he emphasises that forests and other nonhuman entities think and respond to their surroundings. This interaction is part of their sociability within a cosmology overcoming the dichotomy between nature and society as addressed in various ethnographic contributions on the Achuar symbolism and social relation within their environment among others (Descola, 1994; Descola & Pálsson, 2004). Albert et al. (2022) demonstrated that IP territories integrate IP’ social structure.

Scientific evidence has also shown how IP interaction with their territories and ecosystems contribute to an increased biodiversity in their surroundings (Dawson et al., 2021; Fa et al., 2020; Hill, 2011; Pessôa et al., 2023; Ross et al., 2009). Law and Policy literature echoes these results, and highlights how the intersection of biocultural rights, indigenous knowledge, and environmental protection is an avenue for effectively regulating forests and IP protection (Girard et al., 2022; Nikolakis & Hotte, 2020).^12^

Worldwide economic values have prevailed and economic activities in forests have not necessarily been agreed with IP who live therein. Sustainable development mechanisms have sought to balance between economic interests and the need to protect forests and there is evidence of them being partially effective in protecting ecosystems, particularly when inclusive conservation measures are implemented (Maxwell et al., 2020; Raymond et al., 2022). However, sometimes states are sued by lead firms of value chains because with the recognition of protected areas, they have lost the legitimate expectations to exploit these reserves (Cotula & Perrone, 2024; Ünüvar, 2023).^13^ The benefits of protecting ecosystems are not questioned from an Earth system governance perspective, however there is empirical evidence on certain red lines.

First, empirical studies in Latin America did not find clear benefits for IP and their land rights when protected areas are established (De Sa, 2019; Petavratzi et al., 2022; Stetson, 2012). Literature from EJ and anthropology further highlights that land organisation in protected areas is intrinsically related to power relations, often related to postcolonial settings, and sometimes nature conservation projects (e.g. national parks) have resulted in displacement of IP, which has also been documented in Asia and Oceania (Adams & Hutton, 2007; Dowie, 2011; Ganjanapan, 1998; Phongchiewboon et al., 2020).

Second, other studies flag those mechanisms to prevent deforestation, such as the Reducing Emissions from Deforestation and Forest Degradation framework (REDD +), need to integrate IP participation in their design and implementation (Carter et al., 2017; Schroeder, 2010; Schroeder & González, 2019). IP involvement is crucial as some carbon offset policies may result in forced evictions or green grabbing because they may hinder IP right to enjoy their territories or are based in the new form of attributing economic value to forests for climate mitigation purposes (Franco & Borras, 2019; Poirier & Ostergren, 2002). The challenges of implementing mechanisms of the green economy in IP lands and forests are also documented in Peru (Dupuits & Cronkleton, 2020) and Brazil (Garcia et al., 2021) among other.

The discussion on the value attributed to ecosystems has also been translated into influential legal approaches such as ecocentrism, which highlights the intrinsic value of ecosystems (Nash, 1989; White, 2018), the rights of nature that emerge as an alternative to protect ecosystems (Bourgeois-Gironde, 2024; Garver, 2013; Gilbert, 2023), or Mother Earth approaches that interconnect IP interaction with nature as ‘determinants of planetary health’ (Redvers et al., 2022; Viaene, 2022). Some studies argue that this topic has been mainly assessed from a social science perspective and therefore, its effectiveness is inconclusive (Gilbert et al., 2023). Although the rights of nature and those of Mother Earth are used interchangeably, actions taken in the name of nature in IP territories have not always been consulted with them or do not necessarily reflect IP cosmovision (De Froideville & Bowling, 2022; Fa et al., 2020; Petel, 2024; Tănăsescu, 2020). These critical views regarding potential clashes between ecocentrism and IP cosmovision (conceptualised here as BSCA to forest protection) do not align with anthropocentric approaches that attribute essentially economic value to forests (Kotzé & Kim, 2019). However, some ecocentric measures claim to protect the intrinsic value of ecosystems, but may be motivated by their economic value, which is becoming evident in forestry and carbon offset programmes. Other authors have not found empirical evidence of benefits obtained from ecocentric approaches for the protection of ecosystems (Talbot-Jones & Bennett, 2019).

IL plays a significant role in protecting ecosystems and IP, because they frequently cross state borders. However, serious regulatory gaps and limitations have also been found (Kotzé & Kim, 2019; Kulovesi et al., 2019). There has also been evidence that a holistic approach to the protection of IP and forests is needed (Dorough & Wiessner, 2020, p. 414; Eikermann, 2015, p. 12–13) not only because IP’ territories are essential for forest conservation but also because environmental degradation seriously affects IP lands (Garver, 2013; Perkins, 2019).

Finally, scientific evidence shows that many IP are vulnerable and marginalised, and adopting binding mechanisms to protect them requires considering the social-culture-ethnicity-economy nexus (Larsen & Gilbert, 2020; Rodríguez-Garavito, 2011, 2019). Frequently IP claim that their ‘own concepts of their rights to land’ are not incorporated into legal systems (Swepston, 2015, p. 231). Bioculturality emerged primarily to describe how IP rights are essential for biodiversity conservation and related knowledge, mainly linked to issues of access to benefit sharing (ABS) for the use of genetic resources (Girard et al., 2022; Harry et al., 2010; Sajeva, 2015, 2022). However, bioculturality has also been used by courts to indicate the link between IP' rights and their territories and ecosystems where they live (Macpherson et al., 2020), which aligns with the Mother Earth concepts in other countries, such as Ecuador.

This article, building on the literature mainly from ethnoecology, anthropology, or planetary health, considers that the concept of BSCA better reflects the conceptualisation that forests and ecosystems in general are part of IP' cosmovision and society. Restricting the concept of bioculturality to access to benefit-sharing brings the mechanism closer to those who defend a 'sustainable' use of ecosystems, which carries with it the risks identified in the literature regarding overexploitation.^14^

## Which value does international law attribute to forests and IP’ rights?

This section presents the IL and policies regarding forests, IP’ rights and the integration of IP’ cosmovision in forest management. Although three main areas (environmental law, wild law, and the protection of IP’ rights) and some indirect areas (e.g., cultural heritage or trade law), may be relevant, the BSCA connects with emerging areas such as law and ecology, or sustainable development law, particularly since the Rio Declaration^15^ acknowledges that *IP and their communities and other local communities have a vital role in environmental management and development because of their knowledge and traditional practices. States should recognize and duly support their identity, culture and interests and enable their effective participation in the achievement of sustainable development* (Principle 22). This principle is consistent with the scientific evidence described in the literature review. However, more than 30 years after its adoption, IP’ and forest protection are still fragmented and subordinated to policies and projects that prioritise the economic value of forests. This could be explained by the inherent conception of sustainable development proposed since 1972 (Meadows et al., 1974) and reinforced by the Stockholm^16^ (1972) and Rio (1992) Declarations that propose a balance among the triple bottom line of sustainable development: prosperity (the economy), people and the planet (Mother Earth).

### International law on forests

IL on forests has mainly dealt with its economic exploitation.^17^ In general, legal responses to protect soils have failed in preventing land-use change and deforestation. Although soil degradation has been linked to extractive activity and large-scale agriculture, the latter has been justified to guarantee food security. Compared to other elements of the Earth system, such as air and water, only the UN Convention to Combat Desertification (1992) protects soils. The UN Framework Convention on Climate Change (UNFCCC) refers to the need of combating deforestation, but no legal definition has been adopted either. Only the Addendum to the COP 7 to the UNFCCC defines forests and deforestation.^18^

Regarding wild law, the Convention on Wetlands of International Importance (Ramsar Convention, 1971) protects wetlands and if they are in forests, the latter can benefit from this protection, but only because they host wetlands. An evaluation of the effectiveness of the Ramsar Convention fifty years after its adoption shows that wetlands have been seriously affected despite significant growth in the states signing up (Bridgewater & Kim, 2021, p. 3920).

In this scenario, the debate has been about the need for a forest conservation treaty. There is no consensus to adopt a binding treaty to protect forests and combat deforestation. Looking at the arguments in favour and against (Maguire et al., 2020), the conflicting values around forests seem to guide these positions and still, its implementation depends on states’ willingness to enforce them.^19^

At the UN, various non-binding initiatives seek to protect forests.^20^ From the current sustainable development agenda, concretised in the Sustainable Development Goals (SDG), multiple competing targets emerge. Food security (SDG2) has been a crucial topic particularly during the pandemic, armed conflicts, and human-induced disasters, but it may have an impact on access to drinking water (SDG6). States seek to achieve sufficient resources to eradicate poverty (SDG1) while preserving life on land and under water (SGD14 and 15) and controlling global warming (SDG13). Only SDG15 explicitly refers to the need of *protecting, restoring, and promoting sustainable use of terrestrial ecosystems, sustainably manage forests, combat desertification, and halt and reverse land degradation and halt biodiversity loss*.

Another gap of IL is the reluctance to adopt a transnational approach that would enable non-state actors to be participants of the decision-making processes and to hold non-state actors accountable for sustainability related adverse impacts. This has had two major problems. First, IP’ participation in decision-making processes is not enabled, and second, the current duty of states to hold leading firms of value chains accountable for impacts on ecosystems and IP is insufficient to address transboundary impacts (Iglesias Márquez, 2020; Merino & Chinchay, 2022). At the regional level, only the European Union (EU), thanks to its supranational structure, has been able to adopt binding rules^21^ requiring due diligence procedures for some agrifood value chains. Although positive, the timber and the deforestation-free regulations do not cover other impacts such as the release of hazardous materials and waste^22^ and did not refer to IP’ rights, territories, and participation.

### Environmental frameworks that enable a BSCA

This section focuses on how this relationship between IP and forests in which they live has been enabled and protected, so that IP' rights and territories are both protected and integrated into the co-management of forests. The SDGs did not mention the need to include IP in the cosmovision of ecosystems where they live or the need to integrate IP into the ‘sustainable management of forests’. However, other policy documents have made progress in the international recognition of the role of IP in protecting forests.

First, at UNFCCC-COP 24, the Facilitative Working Group (FWG) of the Local Communities and Indigenous Peoples Platform^23^ was created to promote the exchange of best practices related to traditional, indigenous, and local knowledge, enhance IP’ capacity to engage with the UNFCCC process, and facilitate the integration of diverse knowledge systems into international and national actions.^24^ The International Indigenous Peoples' Forum on Climate Change, a caucus for IP participating in the UNFCCC processes, has also called for the inclusion of IP in all proposed climate actions, safeguarding their rights.^25^

Second, the Kunming-Montreal Biodiversity Framework (KMBF)^26^ adopted at COP 15 of the Convention on Biological Diversity (CBD) formulated 23 targets to address the urgent need to halt biodiversity loss, restore ecosystems and protect IP’ rights. This instrument acknowledges the crucial role of IP and local communities *as custodians of biodiversity and as partners in its conservation, restoration and sustainable use* (pg.5). The KMBF mainstreams the protection of IP’ rights and cosmovision and by promoting participatory decision-making processes. Seven of the 23 targets (1,3,5,9,19(f),21,22) recall states to protect IP’ rights from a cultural-sensitive approach, which seems to follow a BSCA. At the CBD-COP 16 IP and people of African descent were recognised as protagonists in biodiversity conservation. It was decided to create a subsidiary body for them under article 8 J of the CBD.^27^

### Protecting forests by protecting IP’ rights

Although several treaties may have an influence on IP’ rights, only three treaties explicitly protect IP rights. ILO-169 has only been ratified by 23 states, 14 of which are Latin American, which may explain the importance of this region in IP’ issues. The CBD and its Nagoya Protocol on Access to Genetic Resources and the Fair and Equitable Sharing of Benefits Arising from their Utilization (The Nagoya Protocol, 2011) recognise the importance of IP in the management of the sustainable exploitation of biodiversity. Although the Paris Agreement timidly refers to IP, no specific measure has been adopted. IP organisations highlighted that according to the UN Declaration on the Rights of Indigenous Peoples^28^ (UNDRIP), IP must be protected by all climate actions.^29^ The UNPFII in turn raised the need for a ‘grievance mechanism’ in the  + Program to hear IP complaints.^30^

This section focuses on two rights, land rights and self-determination (as embodied in Free, Prior and Informed Consent (FPIC) and consultation), without ignoring that other rights may contribute but are less related to the BSCA.

#### Synergies between IP' collective land rights and forests

The ILO-169 (Art.13–9) requires states to respect IP' rights of ownership and possession over their ancestral lands, particularly ‘*the special importance of the cultures and spiritual values of the peoples concerned with their relationship with the lands or territories.*’ (Art.13) However, states can retain ownership of the subsoil, which may affect these territories when the state exploits it, and which has generated multiple conflicts worldwide. This may also conflict with the UN General Assembly (UNGA) Resolution^31^ that recognised peoples’ and not only states’ sovereignty over the ecosystems where they live. However, inexplicably other sustainable development instruments omit the peoples’ sovereignty over natural resources where they live.

IP’ land rights have also been protected by soft law instruments. Several UN non-binding initiatives request states and corporate groups to protect and respect IP’ collective rights, including land rights,^32^ and highlight the importance of respecting IP land rights while protecting forests.^33^ Particularly the UNDRIP (Art.25–8, 30, and 32) acknowledges the intertwined relationship between IP and their ancestral lands in its multiple dimensions, and highlights the need to respect ‘*the customs, traditions, and land tenure systems of the IP[s] concerned*,’ (Art.26). The UNGA also recalls states that when implementing sustainability and eco-friendly practices, they should prioritise IP’ welfare, respect for their ancestral lands, and mitigate the negative environmental consequences of economic activities.^34^

The UNSRIP, EMRIP, and UNPFII, also acknowledge the importance of protecting IP rights when economic activities and climate actions may affect them. UNSRIP has issued reports on green financing,^35^ climate change impact,^36^ investment agreements,^37^ extractive industries,^38^ tourism,^39^ protected areas^40^ and conservation,^41^ where it recommended states and non-state actors (corporations, donors, investors etc.)^42^ to recognise land rights, ensure FPIC and conduct due diligence. EMRIP has also issued advice on land rights^43^ and FPIC.^44^ Finally, UNPFII has issued several recommendations^45^ to states, international, intergovernmental, and regional organisations, fora, donors, foundations, and international funds. The Forum recommended states to assess and report on FPIC,^46^ as part of their duty to protect,^47^ and acknowledge that IP have rights to forests,^48^ among others.

The Inter-American Human Rights System is the regional system that has advanced the most in protecting IP’ collective lands.^49^ The American Declaration on the Rights of Indigenous Peoples^50^ (ADRIP) recognised IP’ rights to traditional forms of property and cultural survival, including the right to land, territory, and resources (Art.XXV). The ADRIP further recognised that IP ‘*have the right to live in harmony with nature and to a healthy, safe, and sustainable environment, essential conditions for the full enjoyment of the rights to life and to their spirituality, cosmovision, and collective well-being*’ (Art. XIX). The Interamerican Commission on Human Rights has also released various reports on indigenous and tribal peoples’ rights over their ancestral lands and natural resources^51^ and the need to protect them from extractive industries.^52^

Three bodies have also held important case law on IP’ collective lands. First, the IACtHR, grounded on the American Convention on Human Rights (Art.21), held that states have three correlated duties: first, to safeguard IP’ genuine ownership, recognise IP’ lands, and grant collective titles to these territories. Second, to refrain from engaging in any activity that might cause adverse impacts on IP’ territories. Third, IP' control and utilisation of their territories and natural resources must be ensured free from any external interference by third parties.^53^ The first IACtHR judgment in relation to the right to communal property^54^ has inspired the African Commission on Human and Peoples’ Rights.^55^

Second, the UN Human Rights Committee (CCPR), following the IACtHR jurisprudence,^56^ highlighted that the International Covenant on Civil and Political Rights (ICCPR, Art.27) in line with the UNDRIP ‘*enshrines the inalienable right of indigenous peoples to enjoy the territories and natural resources that they have traditionally used for their subsistence and cultural identity*.’^57^ Accordingly, the CCPR has held relevant decisions that connect a BSCA to the interaction between IP rights and nature.^58^ This has been corroborated by other UN treaty bodies as well.^59^ Third, the ILO Committee of Experts on the Application of Conventions and Recommendations (ILO-CEACR) has rendered observations in the framework of the applications of ILO-169. For instance, ILO asked India to ensure land rights of tribes and traditional forest dwellers^60^ who faced threats of eviction.^61^ ILO also dealt with representations against states over alleged violations of ILO-169.^62^

#### Indigenous right to self-determination and the free prior and informed consent (FPIC) and consultation

The FPIC, grounded in the right to self-determination and prohibition of discrimination established by the ICCPR, the ICESCR and the International Convention on the Elimination of All Forms of Racial Discrimination,^63^ is another mechanism to ensure IP’ control over their territory. Whether this should be consultation (Article 6(1)(a) of ILO-169) or a stricter requirement of prior consent, which is supported by UNDRIP (Articles 32 and 29(2)) and ADRIP (Article XXIX), has been debated at length. In practice, states have adopted this standard with many variations and deviations, whose analysis goes beyond the purposes of this article. FPIC has been considered as a sort of veto right (Barelli, 2018; Gilbert & Doyle, 2011) that may trigger a negotiation between IP, the state and economic actors. Therefore, FPIC may reinforce IP’ capacity to protect forests within their territories, supported by their right to self-determination (Yaffe, 2018). The Escazu Agreement (art.7.15)^64^ regulating the participatory environmental rights, has also held that when implementing the treaty, states must ensure that they comply with the international obligations on IP’s rights.

The IACtHR has held that ‘*consultation should take place, per the inherent traditions of IP*.’^65^ The UNHRC further highlighted that this obligation is not a mere consultation of the community members.^66^ The ILO Governing Body Committee has requested states to adopt the necessary actions to improve the consultation process concerning the impact of timber concessions (ILO-169, art.6).^67^ The Committee also dealt with IP representations regarding violations of IP rights in relation to the constructions of dams,^68^ highways,^69^ and legislative measures.^70^ In an observation, the ILO-CEACR also flagged concerns about IP impacted by wood resource extraction projects^71^ and recalled that IP should be ‘*consulted before any programmes for the exploration or exploitation of the resources pertaining to their lands are undertaken or permitted*.’^72^

## Discussion

The literature review on topics that connect to a BCSA shows that there is solid scientific evidence that having conflicting values attributed to ecosystems that translate into economic policies may affect IP’ rights and forests independently of the type of economic activity. Even if the extractive or agri-food value chains are the main contributors to deforestation and global warming, conflicts that occur on IP’ lands and forests can also be generated by activities that change land-use, cause forest degradation and deforestation regardless of whether these activities are considered good for the decarbonisation of the planet.

What are good options to protect forests? In the absence of a solid regulatory framework that creates state duties, we visualise three salient approaches: exclude forests from the production system and preserve them as human commons (ecocentrism), reinforce a green growth model (the SDG agenda), and integrate IP’ vision of forests into the policymaking and management of forests that we consider as a BSCA.

The first model may entail the risk of external intervention in fragile states and the privatisation of tropical forests on the grounds that they are humanity's commons. Examples for this can be drawn from the protection of fresh water, which is also part of global commons but is not treated as such (Schroering, 2024). The second model, the current SDG agenda, still gravitates around state sovereignty on natural resources that is incompatible with the Earth system governance. The third model, the BSCA would translate Earth system governance into Mother Earth care. This model is grounded on empirical evidence that shows that under sustainable development models IP’ lands and forests have been seriously affected by transnational adverse impacts described in the updated figures of the PB framework (Gupta et al., 2024; Richardson et al., 2023).

From the legal analysis, the concerns are not only about regulatory gaps. An obstacle to establish a solid Mother Earth care system is that IL is grounded on exclusive state sovereignty to decide on development or conservation projects that may affect IP and the forests where they live. The Mother Earth care encompasses essential limitations because state sovereignty does not always recognise plural nations, which exclude plural views about forests when discussing state duties regarding Earth system governance. Adapting state sovereignty on Mother Earth is not a utopia. Some elements may ground the adoption of BSCA to IL.

First, various instruments refer to the need to protect IP’ rights, lands or territories, and forests. IL has timidly endorsed a plurivision of forests and the need to integrate IP into the decision-making processes and management of ecosystems at risk such as forests. However, this has only been acknowledged in soft law, since the Rio Declaration and more consistently in the KMBF, and in case law. A good principle could be that the KMBF is translated into clear state duties. The CBD-COP16 took positive steps towards the integration of the Mother Earth approach to IL when recognised IP and people of African descent as key actors in biodiversity conservation and decided to establish a subsidiary body for them under the CBD.

Second, the scope of the permanent sovereignty of states over the natural resources located within its jurisdiction is a crucial theme. Even though UNGA Resolution 1803/1962 “Permanent sovereignty over natural resources” recognised peoples and not only states as owners of the ecosystems where they live, other sustainable development instruments skipped the fact that peoples, including IP, also have sovereignty over the natural resources where they live. This approach is incompatible with claims that crucial transboundary ecosystems should be universal commons, as the UN sought to empower states and peoples who live in these ecosystems, given the antecedents of colonisation.

How can we then bridge the gap? From the legal perspective, it seems crucial to engage with the multidisciplinary academic literature that scientifically demonstrates that a monolithic state sovereignty over natural resources is incompatible with the plural governance of Mother Earth. States need to acknowledge that ‘a sustainable use of the natural resources under its jurisdiction’ is not their exclusive prerogative. The BSCA goes beyond keeping a safe space to operate (Rockström et al., 2023). Giving IP sovereignty on their lands encompasses with the scientific evidence that IP cosmovision and way of life are more sustainable than state-based sustainable development policies (Gupta et al., 2024). This Mother Earth care approach also complements Earth system governance because it has shown impressive results.

This is, IL around the Earth system governance needs to incorporate a Mother Earth-centred approach. Two avenues show the path. First, states must enforce the international protection of IP’ rights and lands. This enables the adoption of a BSCA, which also implies the recognition of IP’ collective land rights over the ecosystems in which they live and the co-management according to their social organisation. Although the ILO-169 requires state-parties to recognise and protect IP’ rights to have their own institutions, ways of life and collective lands or territories, this convention has a limited geographical reach. Furthermore, it frequently clashes with development policies involving land, subsoil and mechanisms that protect private property as some state-individual registration has not been enabled to register IP’ collective lands.

Second, states should apply the instruments that align with a Mother Earth caretaking approach that gathers the BSCA scientific results. They would result in a recognition of the sovereignty of IP who live in forests and an integration of IP cosmovision and social institutions in the policymaking and implementation processes regarding forest governance (Zurba et al., 2024). For instance, local cosmologies and social interactions need to be integrated into the economic cost–benefit analysis of development policies. A point of departure could be to implement the KMBF in a way that results in clear state duties that acknowledge the shared sovereignty over the natural resources with the IP living in these ecosystems.

The main challenge for IL for which BSCA could assist is to combine diverse, and sometimes opposing visions into a single legal framework. BSCA could assist in going beyond the transposition of indigenous visions to legislation. The regulatory initiatives need to be apt to be applicable to IP’ societies. Consequently, a pure legal approach or adopting more rules is insufficient to create a dialogue among and within societies. The application of social sciences conceptual frameworks and methodologies can contribute to constructing a better avenue for the recognition of IP as forests trustees (owners).

This pluralist view of forests is compatible with the arguments in favour of transforming IL into transnational law, where non-state actors have legal recognition to participate in decision making processes but where also corporations and development institutions can be held accountable for transboundary ecological harms (Zurba et al., 2024). This is because not only do states decide on development policies, but many transnational corporations play a significant role in the exclusive economic valuation of forests, either to extract their resources or to preserve them to claim emission rights for their industries.

## Conclusions

Forests have a vital role in the survival and wellbeing of the Planet and of IP who live there. So far IL has not provided an adequate answer to the urgency of protecting forests, which is well documented in the update of the PB framework. If scattered provisions, such as REDD + , aimed at the preservation of the forests, they are not adopted with the participation of IP and sometimes they even cause serious harms to them and their forests. Although IL recognises IP’ rights and relevance for humanity, it has not been able to secure an effective framework for enforcing their important agency in forest conservation. The limited recognition of collective rights to lands as compared to the legal protection of individual private property is one shortcoming of IL. As IP’ societies are usually constructed differently from those acknowledged by state law, a BSCA, in which a holistic view of IP’ society and cultural rights is adopted, could enable the linking of IP rights and forest protection to progressively reshape an international legal framework that seeks a Mother Earth care approach.

Based on a narrative multidisciplinary literature review that complemented a gap analysis of the current IL and policy on the interaction between IP and the forests they live in, this article contributes with three findings. First, the convergence of scientific evidence on the fact that IP are undoubtedly essential actors in the conservation of the ecosystems in which they live. Secondly, it shows the urgent need to reformulate the permanent sovereignty of states over their natural resources, not to weaken their independence vis-à-vis powerful states, but to require them to recognise the plurality of their inhabitants and the need to prioritise the protection of key endangered ecosystems by integrating IP into their management and recognising their rights over them. Third, the recognition of the multi-ethnic character of world populations requires to rethink the state centred model of IL because without this recognition of plural visions, Mother Earth will collapse.

## Abbreviations

ABS: Access to Benefit Sharing
ADRIP: American declaration on the rights of indigenous peoples
BSCA: Bio-socio-cultural approach
CBD: Convention on biological diversity
CCPR: The Un Human Rights Committee
CESCR: The UN Committee on Economic, Social and Cultural Rights
COP: Conference of the parties
EJ: Environmental justice
EMRIP: Expert mechanism on the rights of indigenous peoples
EU: European Union
FAO: Food and Agriculture Organization of the United Nations
FPIC: Free, Prior, and Informed Consent
FWG: Facilitative working group
GHGs: Greenhouse gases
IACtHR: Inter-American court of human rights
ICCPR: International covenant on civil and political rights
ICESCR: International covenant on economic social and cultural rights
IEL: International environmental law
IL: International law
ILO: International Labour Organisation
ILO-CEACR: ILO committee of experts on the application of conventions and recommendations
IP: Indigenous peoples
ILO-169: ILO convention 169 on indigenous and tribal peoples
KMBF: Kunming-Montreal biodiversity framework
LCIPP: Local community and indigenous Peoples platform
PB: Planetary boundaries
REDD +: Deforestation and forest degradation framework
SDG: Sustainable development goals
UN: United Nations
UN-CESCR: UN committee on economic social and cultural rights
UNDRIP: UN Declaration on the rights of indigenous Peoples
UNSRIP: Special rapporteur on indigenous Peoples
UNSRIP: UN Special rapporteur on indigenous Peoples
UN-ECLAC: UN Economic commission for Latin America and the Caribbean
UNFCCC: UN Framework convention on climate change
UNGA: UN General Assembly
UNHRC: UN Human rights council
UNPFII: UN Permanent forum on indigenous issues
VGGT: Voluntary Guidelines on the Responsible Governance of Tenure of Land, Fisheries and Forests in the Context of National Food Security (FAO).

## Footnotes

1 International Labour Organisation (ILO) Convention 169 on Indigenous and Tribal Peoples – (ILO-169) (Art.1) identifies IP as ‘peoples in independent countries who are regarded as indigenous on account of their descent from the populations which inhabited the country, or a geographical region to which the country belongs, at the time of conquest or colonisation or the establishment of present state boundaries and who, irrespective of their legal status, retain some or all of their own social, economic, cultural and political institutions’. The term IP also includes First Nations, Aboriginal people, and the terminology varies from state to state.

2 Although figures on the actual or potential contribution of IP to biodiversity conservation may vary, the crucial role of IP in protecting the areas where they live is widely recognised (Fernández-Llamazares et al., 2024; Gupta et al., 2024).

3 See the project UN in Harmony with Nature, http://harmonywithnatureun.org/chronology/

4 UNSRIP, UN Doc.: A/HRC/4/32, 27.2.2007.

5 In IL social rights refer to other categories of rights (e.g. labour and health rights). See the International Covenant on Economic Social and Cultural Rights (ICESCR, 1986).

6 Land and territory are used in diverse manners across various disciplines (Anthias, 2021). ‘Land’ refers to the physical space where individuals or communities, including IP may have rights, including on the natural resources located in these lands (Ding et al., 2016). The notion of ‘territory’ might also vary across historical and geographical contexts and does not have a single, universal definition (Elden, 2010). UN documents do not explicitly distinguish these concepts and frequently they mention them together as “land, territory and resources” (UNPFII, UN Doc.: E/2024/43 E/C.19/2024/8, para.6). However, there is a gradual preference in the jurisprudence of the Inter-American Court of Human Rights (IACtHR) to use the concept of ancestral territory to refer to IP lands, however, it is not used in an exclusive manner to refer to their collective lands. In this article, we treat ‘land’ and ‘territory’ as interchangeable, following the legal definition provided by (ILO-169, Art. 13.2): ‘*the use of the term lands […] shall include the concept of territories, which covers the total environment of the areas which the peoples concerned occupy or otherwise use*.’.

7 EJ intersects several social sciences, such as legal anthropology, political ecology, ecological economics, and other socio-legal studies. We group them together because this literature generally documents the unequal distribution of access to natural resources and the unequal burden generated by environmental damage.

8 This literature was found by previous reviews conducted by the authors from their areas of expertise (sustainable development law, law and ecology, IL, anthropology, and planetary health), complemented with a search, using as keywords: BSCA, IP, anthropocentric, biocultural and ecocentric approaches to forests.

9 We searched for thematic documents available at 1. UN level, particularly the Special Rapporteur on Indigenous Peoples (UNSRIP) and the Expert Mechanism on the Rights of Indigenous Peoples (EMRIP). For the UN Permanent Forum on Indigenous Issues (UNPFII), we filtered the recommendations by the keyword ‘member states’. 2. The Thematic Rapporteurs related to IP issued by the Inter-American Commission on Human Rights, and 3. Conference of the Parties (COP) to the UNFCCC database using the keywords ‘IP’ and ‘forests’.

10 Two ILO mechanisms were consulted: representation (Art.24 of ILO Convention) and observations.

11 In Nordic countries, Sámi people have a distinct sociocultural history (Broderstad, 2011; Kent, 2018) within their countries and relationship to environment (Helander-Renvall, 2010) and have also been affected by extractives.

12 Some UN policy documents also acknowledge the benefits of involving IP in forest governance in Latin America (FAO & Filac, 2021).

13 Cf. Eco Oro Minerals Corp. v. Republic of Colombia, International Center for Settlement of Investment Disputes (ICSID) Case No. ARB/16/41.

14 ABS has also been used when IP seek to obtain economic benefits from activities conducted in their territories, including extractives (Britcyna, 2019; Wilson, 2019). Whether IP’s expectations regarding ABS are fulfilled, has not been evaluated (Wall & Haslam McKenzie, 2023). ABS is relevant for IP but it mainly seeks to guarantee access to commercial benefits when natural resources and ancestral knowledge are exploited. Therefore, the topic goes beyond the scope of this article.

15 UN Rio Declaration on Environment and Development, UN A/CONF.151/26 (Vol.I) https://www.un.org/en/development/desa/population/migration/generalassembly/docs/globalcompact/A_CONF.151_26_Vol.I_Declaration.pdf

16 Declaration of the United Nations Conference on the Human Environment https://documents.un.org/doc/undoc/gen/nl7/300/05/pdf/nl730005.pdf

17 International Tropical Timber Agreement Geneva, 18.11.1983. UNTS, Vol.1393, p.67; International Tropical Timber Agreement, Geneva, 26.11.1994, UNTS 81; International Tropical Timber Agreement, Geneva, 27.01.2006, UN Doc. TD/TIMBER.3/12.

18 See FCCC/CP/2001/13/Add.1, Decision 11/CP.7, ANNEX *Definitions, modalities, rules and guidelines relating to land use, land-use change and forestry activities under the Kyoto Protocol*. FCCC/CP/2001/13/Add.1 (unfccc.int).

19 See also Abdenur, A. ‘A Global Forest Treaty Is Needed Now’, *PassBlue*, 10.12.2020, at https://www.passblue.com/2020/12/10/a-global-forest-treaty-is-needed-now/

20 See the *Authoritative Statement of Principles for a Global Consensus on the Management, Conservation and Sustainable Development of All Types of Forests* (see A/CONF.151/26/Rev.1 (Vol. I)) adopted at the UN Conference on Environment and Development (1992). See other initiatives adopted in the UN Forum on Forests (https://www.un.org/esa/forests/index.html) and on sustainable soil at the Food and Agriculture Organization (FAO) (https://www.fao.org/policy-support/tools-and-publications/resources-details/en/c/1027927/).

21 See Regulation (EU) No 995/2010 of 20.10.2010 laying down the obligations of operators who place timber and timber products (*OJ L 295, 12.11.2010, p. 23–34)* and the substitute Regulation (EU) 2023/1115 of 31.05.2023 on the making available on the Union market and the export from the Union of certain commodities and products associated with deforestation and forest degradation (OJ L 150, 9.6.2023, p.206–47).

22 These topics are regulated by several conventions, e.g.: the Basel Convention on the Control of Transboundary Movements of Hazardous Wastes (1989), the Stockholm Convention on Persistent Organic Pollutants (2001), the Rotterdam Convention on the FPIC Procedure for Certain Hazardous Chemicals and Pesticides in International Trade (2004) and the Minamata Convention on Mercury (2013).

23 It was established to operationalise the Local Community and to Indigenous Peoples Platform (LCIPP) and to implement knowledge enhancement, capacity building for engagement, and the development of climate change policies and actions. The Working Group has 14 representatives equally divided between Party representatives and indigenous organisations.

24 Decision 2/CP.24 A COP 24 at https://unfccc.int/documents/65126

25 GST TD1.3 Opening Plenary, at https://unfccc.int/documents/630999

26 CBD/COP/DEC/15/4 of 19.12.2022. at https://www.cbd.int/article/cop15-cbd-press-release-final-19dec2022

27 UN News: *COP16: Landmark biodiversity agreements adopted*, 3.11.2024. At https://news.un.org/en/story/2024/11/1156456

28 UN Doc.: A/RES/61/295 of 13.9.2007.

29 International IP' Forum on Climate Change-GST TD1.3 Opening Plenary. https://unfccc.int/sites/default/files/resource/GST_Opening_Plenary_IIPFCC.pdf

30 UN Doc.: E/C.19/2023/7.

31 UNGA Resolution 1803 (XVII) on the “Permanent Sovereignty over Natural Resources”, 14.12.1962 https://legal.un.org/avl/ha/ga_1803/ga_1803.html

32 Cf. 1) The UNDRIP, 2) The UN Declaration on the Rights of Peasants and Other People Working in Rural Areas (2018), and, 3) the FAO's Voluntary Guidelines on the Responsible Governance of Tenure of Land, Fisheries and Forests in the Context of National Food Security (VGGT, 2012).

33 The Committee on Economic, Social and Culture Rights (CESCR), General Comment 22/2016 highlighted that states should avoid climate change mitigation policies like large-scale reforestation that result in land grabbing and affecting IP.

34 UN Doc.: A/77/460.

35 UN Doc.: A/HRC/54/31.

36 UN Doc.: A/HRC/36/46.

37 UN Doc.: A/HRC/33/42.

38 UN Doc.: A/HRC/33/42.

39 UN Doc.: A/78/162.

40 UN Doc.: A/77/238.

41 UN Doc.: A/71/229.

42 UN Docs.: A/HRC/54/31, A/HRC/36/46, A/HRC/33/42, A/HRC/24/41, A/78/162.

43 UN Doc.: A/HRC/45/38.

44 UN Doc.: A/HRC/39/62.

45 See UN UNPFII Recommendations Database https://unpfii.desa.un.org/recommendations

46 UNPFII, 10–2021, para.158, E/2010/43, E/C.19/2010/15 https://www.un.org/esa/socdev/unpfii/documents/E_2010_43_EN.pdf.

47 UNPFII 21-2022, para.5, E/2022/43, E/C.19/2022/11, https://documents.un.org/doc/undoc/gen/n22/355/75/pdf/n2235575.pdf?token=tGOgGAh3wHqDAtFZST&fe=true

48 UNPFII 10-2011, para.155, E/2010/43, E/C.19/2010/15 https://www.un.org/esa/socdev/unpfii/documents/E_2010_43_EN.pdf.

49 See IACtHR, Kaliña and Lokono Peoples v Suriname, Judgement of 25.11.2015, paras.122-8; IACtHR, Mayagna (Sumo) Awas Tingni Community v. Nicaragua, Judgement of 31.08.2000, paras.148-51. See also IACmHR, Comunidad Yanomami, Resolución 12/85 and (Dorough & Wiessner, 2020, p. 422).

50 AS Doc.: AG/RES. 2888 (XLVI-O/16).

51 See OEA/Ser.L/V/II. Doc. 56/09.

52 See OEA/Ser.L/V/II. Doc. 47/15.

53 IACtHR, Indigenous Communities of the Lhaka Honhat (Our Land) Association v. Argentina. Judgment of 6.2.2020.

54 IACtHR, Mayagna (Sumo) Awas Tingni Community v. Nicaragua, Judgement of 31.8.2001.

55 See ACHPR, Center for Minority Rights Development (Kenya) v. Kenya, 276/2003, 4.2.2010.

56 See IACtHR, Mayagna (Sumo) Awas Tingni Community v. Nicaragua and IACtHR, Saramaka People v. Suriname, judgment of 28.11.2007.

57 See CCPR, Pereira and members of the Campo Agua’ẽ indigenous community v. Paraguay CCPR/C/132/D/2552/2015, 21.09.2022. Also see UN Human Rights Committee on Civil and Political Rights (CCPR) General Comment 23(1994): Article 27, CCPR/C/21/Rev.1/Add.5 at para.7.

58 See e.g. CCPR, Angela Poma Poma v. Peru, Communication No. 1457/2006, 29.7.2009 and UNHRC, Billy and others v. Australia, CCPR/C/135/D/3624/2019.

59 See CCPR, Mahuika et al. v. New Zealand, CCPR/C/70/D/547/1993; Äärelä and Näkkäläjärvi v. Finland, CCPR/C/73/D/779/1997; Jarle Jonassen and members of the Riast/Hylling reindeer herding district, CCPR/C/76/D/942/2000; Howard v. Canada, CCPR/C/84/D/879/1999; Kalevi Paadar, Eero Paadar and his family, Veijo Paadar, and Kari Alatorvinen and his family v. Finland, CCPR/C/110/D/2102/2011; CESCR, Lars-Anders Ågren et al. v. Sweden, CERD/C/102/D/54/2013.

60 ILO-CEACR, Observation – India, 109th ILC session (2021), at https://normlex.ilo.org/dyn/normlex/en/f?p=1000:13100:0::NO:13100:P13100_COMMENT_ID,P11110_COUNTRY_ID,P11110_COUNTRY_NAME,P11110_COMMENT_YEAR:4049340,102691,India,2020

61 See UNGA (17.7.2019) *Adequate housing as a component of the right to an adequate standard of living, and the right to non-discrimination in this context*, UN Doc.: A/74/183.

62 E.g. Bolivia was asked to protect IP rights to land and natural resources, and to engage in consultations with them when creating conservation units in their territories. See, Report of the Committee set up to examine the representation alleging non-observance by Bolivia of ILO-169, 1999, at https://normlex.ilo.org/dyn/normlex/en/f?p=1000:50012:0::NO:50012:P50012_COMPLAINT_PROCEDURE_ID,P50012_LANG_CODE:2507170,en:NO

63 See also UN HRC (2018) Study of the Expert Mechanism on the Rights of Indigenous Peoples, Free, Prior, and Informed Consent: A Human Rights-Based Approach. UN Doc. A/HRC/39/62, para.3; IACtHR, Case of the Saramaka People v. Suriname, judgment of 28.11.2007, para.133 s.

64 UN Economic Commission for Latin America and the Caribbean (ECLAC) Regional Agreement on Access to Information, Public Participation and Justice in Environmental Matters in Latin America and the Caribbean, 2018.

65 See IACtHR, Case of the Mayagna (Sumo) Awas Tingni Community v. Nicaragua, judgment of 31.8. 2001, para.180.

66 See UNHRC, Angela Poma Poma v. Peru, Communication No. 1457/2006, 29.7.2009. See also (Cabrera Ormaza, 2018, p. 55–62; Erueti, 2022, p. 160).

67 ILO-Governing Body Committee Report on non-observance by Brazil of ILO-169, 2009, https://normlex.ilo.org/dyn/normlex/en/f?p=1000:50012:0::NO:50012:P50012_COMPLAINT_PROCEDURE_ID,P50012_LANG_CODE:2507317,en:NO

68 See ILO-Governing Body Committee Report on non-observance by Peru of ILO-169, (https://normlex.ilo.org/dyn/normlex/en/f?p=NORMLEXPUB:50012:0::NO::P50012_COMPLAINT_PROCEDURE_ID,P50012_LANG_CODE:2507473,en).

69 See ILO-Governing Body Committee Report on non-observance by Mexico of the ILO-169 (https://normlex.ilo.org/dyn/normlex/en/f?p=1000:50012:0::NO:50012:P50012_COMPLAINT_PROCEDURE_ID,P50012_LANG_CODE:2507302,en:NO).

70 See ILO-Governing Body Committee Report on non-observance by Brazil of the ILO-169 (https://normlex.ilo.org/dyn/normlex/en/f?p=1000:50012:0::NO:50012:P50012_COMPLAINT_PROCEDURE_ID,P50012_LANG_CODE:2507317,en:NO).

71 CEACR ILO, Direct request – Bolivia, 109th ILC session (2021), at https://normlex.ilo.org/dyn/normlex/en/f?p=1000:13100:0::NO:13100:P13100_COMMENT_ID,P11110_COUNTRY_ID,P11110_COUNTRY_NAME,P11110_COMMENT_YEAR:4023313,102567,Bolivia%20(Plurinational%20State%20of),2019

72 CEACR ILO, Observation – Paraguay, 107th ILC session (2018), at https://normlex.ilo.org/dyn/normlex/en/f?p=1000:13100:0::NO:13100:P13100_COMMENT_ID,P13100_COUNTRY_ID:3341595,102796
